# Skeletal maturation in different anteroposterior and vertical skeletal growth patterns in female subjects

**DOI:** 10.1007/s00056-022-00389-4

**Published:** 2022-04-06

**Authors:** Mostafa M. El-Dawlatly, Ahmed Y. Abdelghaffar, Juan Martin Palomo, Yehya A. Mostafa

**Affiliations:** 1 Department of Orthodontics and Dentofacial Orthopedics, Faculty of Oral and Dental Medicine, Cairo University, Cairo, Egypt; 2 Orthodontics Department, Faculty of Oral and Dental Medicine, Cairo University, Cairo, Egypt; 3 Department of Orthodontics, Craniofacial Imaging Center, Case Western Reserve University, Cleveland, OH USA; 4 Department of Orthodontics and Dentofacial Orthopedics, Faculty of Oral and Dental Medicine, Future University, Cairo, Egypt; 5 8/1 5th section, Nerco, Degla, Maadi, Cairo, Egypt

**Keywords:** Facial height, Growth modification, Skeletal patterns, Cervical vertebrae maturation index, Skeletal deformities, Gesichtshöhe, Modifikation des Wachstums, Skelettale Muster, Reifungsindex der Halswirbelsäule, Skelettale Deformitäten

## Abstract

**Purpose:**

The aim of the present work was to study the sequence of skeletal maturation in the various anteroposterior and vertical skeletal growth patterns and to detect whether differences existed between them.

**Methods:**

Cephalograms of 861 growing and adolescent female patients were traced to categorize the subjects into 9 skeletal patterns. Each subject was assigned a skeletal maturational stage. Analysis of variance (ANOVA) followed by Bonferroni test were used to detect differences in the onset of the three growth stages (prepubertal, pubertal and postpubertal) between the 9 groups. The same statistical methods were used to detect differences between the mean ages at the three growth stages within each group.

**Results:**

No statistically significant differences were found between the mean ages of pubertal and postpubertal growth stages between the 9 skeletal patterns. However, class III growers had a significantly earlier onset of prepubertal growth (10.25 ± 1.56 years) when compared to that of class II high angle cases (11.11 ± 1.67 years; *P* < 0.01). Also, significant differences were found between the mean ages at the three growth stages within the groups.

**Conclusion:**

A map was created defining the sequence of skeletal maturation for each skeletal growth pattern. This map defines clinically relevant differences in the starting time points and the optimum intervals of growth modification for each skeletal growth pattern.

## Abstract (de)

**Zielsetzung:**

Ziel der vorliegenden Arbeit war es, die Abfolge der Skelettreifung bei den verschiedenen anteroposterioren und vertikalen skelettalen Wachstumsmustern zu untersuchen und festzustellen, ob Unterschiede zwischen ihnen bestehen.

**Methoden:**

Anhand von ihren Kephalogrammen wurden 861 heranwachsende und jugendliche Patientinnen 9 verschiedenen skelettalen Wachstumsmustern zugeordnet. Jeder Probandin wurde ein Skelettreifungsstadium zugewiesen. Mittels Varianzanalyse (ANOVA) und anschließendem Bonferroni-Test wurden Unterschiede zwischen den 9 Gruppen hinsichtlich des Beginns der 3 Wachstumsstadien (vorpubertär, pubertär und postpubertär) festgestellt. Die gleichen statistischen Methoden wurden angewandt, um Unterschiede zwischen dem mittleren Alter in den 3 Wachstumsstadien innerhalb jeder Gruppe festzustellen.

**Ergebnisse:**

Es wurden keine statistisch signifikanten Unterschiede zwischen den mittleren Alterswerten der pubertären und postpubertären Wachstumsstadien zwischen den 9 skelettalen Mustern festgestellt. Allerdings setzte bei den in der Klasse III wachsenden Kindern das vorpubertäre Wachstum signifikant früher ein (10.25 ± 1.56 Jahre) als bei den Kindern mit hohem Winkel in der Klasse II (11.11 ± 1.67 Jahre; *p* < 0,01). Es wurden auch signifikante Unterschiede zwischen dem mittleren Alter in den 3 Wachstumsstadien innerhalb der Gruppen festgestellt.

**Schlussfolgerung:**

Es wurde eine Übersicht erstellt, die die Abfolge der Skelettreifung für jedes Skelettwachstumsmuster definiert. Diese Übersicht definiert klinisch relevante Unterschiede bei den Anfangszeitpunkten und den optimalen Intervallen der Wachstumsmodifikation für jedes skelettale Wachstumsmuster.

## Introduction

The optimum timing of growth modification in patients with skeletal anteroposterior or vertical discrepancies has been established to be during the pubertal growth spurt [1]. The onset of this pubertal growth spurt was reported to vary between different ethnic groups, genders, populations and even between social standards [2–4]. Moreover, the prepubertal growth stage was reported in many studies to be as crucial as the pubertal spurt. Growth modification of some skeletal deformities, especially those where the maxillary arch growth is to be controlled, seems to end up with better results when it takes place during this stage [5]. In addition, some interceptive and preventive procedures and habit breaking protocols heavily rely on the timing of the prepubertal growth stage.

It was reported that subjects with skeletal deep and open bites would reach their pubertal growth spurt at different time points. Hence, it has been recommended that growth modification should start earlier in growing subjects having an increased lower facial height [6].

Moreover, with variable onsets for pubertal or prepubertal growth stages, the optimum timing of growth modification for different skeletal patterns could differ. In addition, different growth patterns might have variations in the time needed to reach the postpubertal growth stage which could directly affect the length of the retention period required after growth modification therapy.

Many studies recommended variable timing for growth modification for the various anteroposterior skeletal patterns. It has been reported that skeletal class III subjects have a longer pubertal growth spurt interval than class I growing patients [7]. The peak pubertal growth duration was also found to be, on average, 4 months shorter in class II subjects and 6 months longer in class III subjects when compared to class I controls [8]. Growth modification of growing class III subjects has been reported to be better attained early in the prepubertal growth stages [5], while class II growth modification therapy, especially for subjects having mandibular deficiency, has been reported to be better achieved late during the pubertal growth spurt [4, 9]. Moreover, it was proven that mandibular growth in class II patients differs significantly from that of control patients having normal occlusion [10].

In addition, defining differences in the timing of growth between different anteroposterior skeletal classes, regardless of their vertical growth pattern, would not give us a complete image of the accurate onsets of the growth stages for the individual patient.

In the current study, the main aim was to detect the differences in the onsets of the prepubertal, pubertal and postpubertal stages between the various skeletal patterns. In addition, we assessed the progress of the skeletal maturation sequences in different skeletal patterns.

## Materials and methods

Sample size calculation was done using the Power and Sample Size Program (version 3.1.2 for Windows XP). It showed that a minimum sample size of 75 patients would be necessary within each group to detect a significant difference in maturation between the 9 skeletal patterns. The power was set at 80% and the significance level at 0.05. The sample sizes were set to be more than 90 subjects within each subgroup. This aimed to ensure that the study power was achieved and to compensate for any expected poor radiographs. The study was approved by the ethics committee of the Faculty of Dentistry, Cairo University with a reference number of 12,521.

Thus, the sample comprised pretreatment lateral cephalograms of 861 growing and adolescent female patients that were filtered from 3870 radiographs from the pretreatment records saved at the outpatient clinic computer of the Department of Orthodontics of two different universities. However, it was ensured that both Universities used the same cephalometric radiography machine to avoid different magnification. The subjects were aged 8–16 years, and their selection was based on the following inclusion criteria: (1) clear sharp cephalometric radiograph, (2) clear visibility of all cervical vertebrae, (3) no previous orthodontic treatment. Exclusion criteria comprised the following: (1) patients having syndromes, (2) radiographs which were not taken in centric occlusion, (3) radiographs of patients with ongoing orthodontic treatment.

**Fig. 1:**
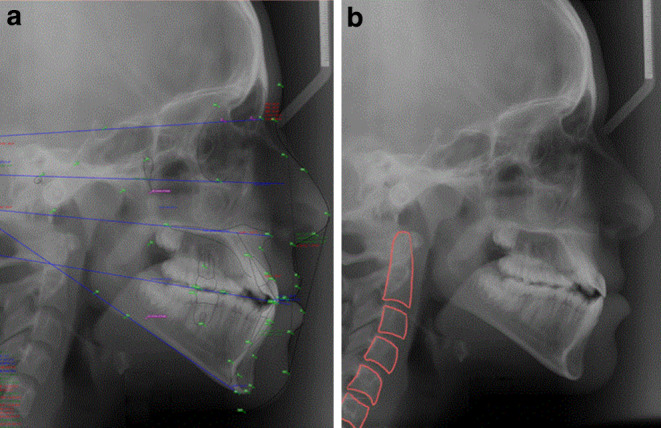
**a** Digital tracing of the cephalograms using OrisCEPH software. **b** Tracing of the cervical vertebrae to assess the cervical vertebrae maturation index (CVMI) stage **a** Digitale Verfolgung der Kephalogramme mit der OrisCEPH-Software. **b** Durchzeichnen der Halswirbelsäule zur Beurteilung des CVMI(„cervical vertebrae maturation index“)-Stadiums

A total of 6 cephalometric skeletal measurements (Table 1) were used in this study. They included three anteroposterior and three vertical measurements. These measurements were obtained using OrisCEPH R × 3 (version 7.2.3) software (Fig. 1a). The cervical vertebrae maturation index (CVMI) of each cephalogram was determined via manual tracing of the cervical vertebrae ([11]; Fig. 1b). The assessor was blinded from the originally traced lateral cephalogram and all the tracing outcomes. For the sake of inter- and intraobserver reliability, cephalometric and cervical vertebrae measurements of 300 cephalograms were repeated by the same observer at another occasion (with a 2-week interval) and by another professional observer.

**Table 1:** Anteroposterior and vertical cephalometric measurement done using the OrisCEPH software Anteroposteriore und vertikale kephalometrische Messung mit der OrisCEPH-Software

Anteroposterior measurements		Vertical measurements	
Measurement	Normal values	Measurement	Normal values
ANB	2–4°	MMPA	22–28°
A–B difference	2–4 mm	SN/MP	29–39°
Wits appraisal	1 to −1 mm	FMPA	22–30°

The age of each subject included in the study was determined by the records found on the cephalometric x‑rays. For each subject, the distinct growth maturation stage and growth pattern (both vertical and anteroposterior) were determined. The anteroposterior and vertical growth patterns were determined if 2 of the 3 anteroposterior and vertical skeletal measurements confirmed a certain class. In addition, after establishing the vertical relation according to the cephalogram, the final growth pattern of the patient was confirmed by inspecting the frontal and lateral photos taken at rest. This led to the allocation of the patients to 9 different skeletal growth patterns:

- Class I horizontal growers: 96 cases
- Class I normal growers: 95 cases
- Class I vertical growers: 94 cases
- Class II horizontal growers: 95 cases
- Class II normal growers: 98 cases
- Class II vertical growers: 98 cases
- Class III horizontal growers: 91 cases
- Class III normal growers: 98 cases
- Class III vertical growers: 96 cases

Stages CS1 and CS2 of the cervical vertebrae maturation index were considered to represent the prepubertal stage. Stages CS3 and CS4 were considered the pubertal stage, while stages CS5 and CS6 were considered the postpubertal stage [4].

The primary outcome of the study was the detection of possible differences between the onsets of the 3 growth stages (prepubertal, pubertal, postpubertal) between the 9 skeletal patterns. The secondary outcome was the assessment the skeletal maturation sequence within each group.

## Statistical analysis

Statistical analysis was performed using SPSS software (version 22.0; IBM, Armonk, NY, USA) for Windows. For both primary and secondary outcomes, one-way analysis of variance (ANOVA) followed by Bonferroni method for multiple comparisons was applied for hypothesis testing of equality of the several group means. A statistically significant one-way ANOVA gives an unspecific indication of the existence of differences between the studied groups. But, in order to define the specific groups between which differences existed, the Bonferroni method was applied. Inter- and intraobserver reliability analysis was carried out using the Dahlberg error (DE) and relative Dahlberg error (RDE) as well as the concordance correlation coefficient (CCC).

## Results

For a clear interpretation of the results, three main sectors (interobserver and intraobserver reliability, intergroup analysis, and intragroup analysis) were considered.

Excellent interobserver and intraobserver reliability was detected. RDE did not exceed 10% and CCC values were recorded between 0.991 and 1.

**Fig. 2:**
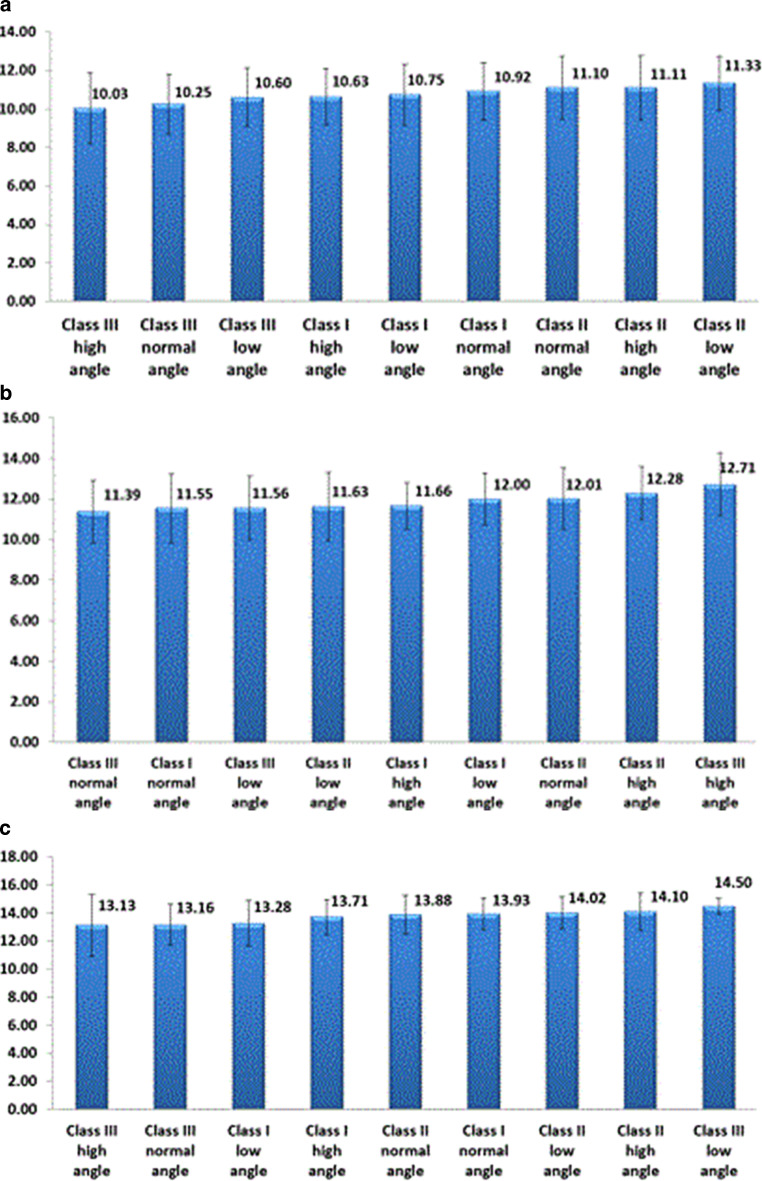
Bar charts showing the mean ages of the 9 skeletal patterns at the **a** prepubertal stage, **b** pubertal stage and **c** postpubertal stage Balkendiagramme mit dem mittleren Alter der 9 skelettalen Muster im **a** präpubertären, **b** pubertären und **c** postpubertären Stadium

Intergroup analysis (within each growth stage the mean ages of the subjects were compared among the 9 different skeletal growth patterns) was divided into three sections. The first section compromised the prepubertal growth stage (10.03–11.33 mean ages; Table 2; Fig. 2*). *The ANOVA test showed statistically significant (*P* < 0.01) group differences. The Bonferroni test detected that class III normal angle cases had a significantly earlier onset of prepubertal growth when compared to that of class II high angle cases (*P* < 0.01). The second section comprised the pubertal growth spurt (11.39–12.71 mean ages; Fig. 2). No statistically significant differences were found for the pubertal growth stage mean ages between the 9 skeletal patterns. The third section was the postpubertal growth stage (13.13–14.5 mean ages; Fig. 2). No statistically significant differences were found for the postpubertal growth stage mean ages between the 9 skeletal patterns.

**Table 2:** Differences between the mean ages of the 9 skeletal pattern groups at the prepubertal growth stage assessed by Bonferroni method for intergroup statistics Unterschiede zwischen den mittleren Alterswerten der 9 Gruppen von skelettalen Mustern in der präpubertären Wachstumsphase, ermittelt anhand der Bonferroni-Methode für Intergruppenstatistiken

Skeletal pattern	Skeletal pattern	MD	SE	*P* value
Group 1	Group 2	−0.17	0.61	1.00000
Group 1	Group 3	0.12	0.65	1.00000
Group 1	Group 4	−0.58	0.78	1.00000
Group 1	Group 5	−0.35	0.60	1.00000
Group 1	Group 6	−0.36	0.58	1.00000
Group 1	Group 7	0.15	0.92	1.00000
Group 1	Group 8	0.50	0.61	1.00000
Group 1	Group 9	0.72	0.67	1.00000
Group 2	Group 3	0.29	0.38	1.00000
Group 2	Group 4	−0.41	0.58	1.00000
Group 2	Group 5	−0.18	0.28	1.00000
Group 2	Group 6	−0.19	0.25	1.00000
Group 2	Group 7	0.32	0.75	1.00000
Group 2	Group 8	0.68	0.30	0.96665
Group 2	Group 9	0.89	0.42	1.00000
Group 3	Group 4	−0.70	0.62	1.00000
Group 3	Group 5	−0.47	0.36	1.00000
Group 3	Group 6	−0.48	0.33	1.00000
Group 3	Group 7	0.03	0.79	1.00000
Group 3	Group 8	0.38	0.37	1.00000
Group 3	Group 9	0.60	0.47	1.00000
Group 4	Group 5	0.23	0.56	1.00000
Group 4	Group 6	0.22	0.55	1.00000
Group 4	Group 7	0.73	0.90	1.00000
Group 4	Group 8	1.09	0.58	1.00000
Group 4	Group 9	1.30	0.64	1.00000
Group 5	Group 6	−0.01	0.21	1.00000
Group 5	Group 7	0.50	0.74	1.00000
Group 5	Group 8	0.85	0.27	0.05992
Group 5	Group 9	1.07	0.39	0.24347
Group 6	Group 7	0.51	0.73	1.00000
Group 6	Group 8	0.86825	0.24	0.01422*
Group 6	Group 9	1.08	0.37	0.14609
Group 7	Group 8	0.35	0.75	1.00000
Group 7	Group 9	0.57	0.80	1.00000
Group 8	Group 9	0.21	0.41	1.00000

*Statistically significant

*MD* mean difference, *SE* standard error

**Fig. 3:**
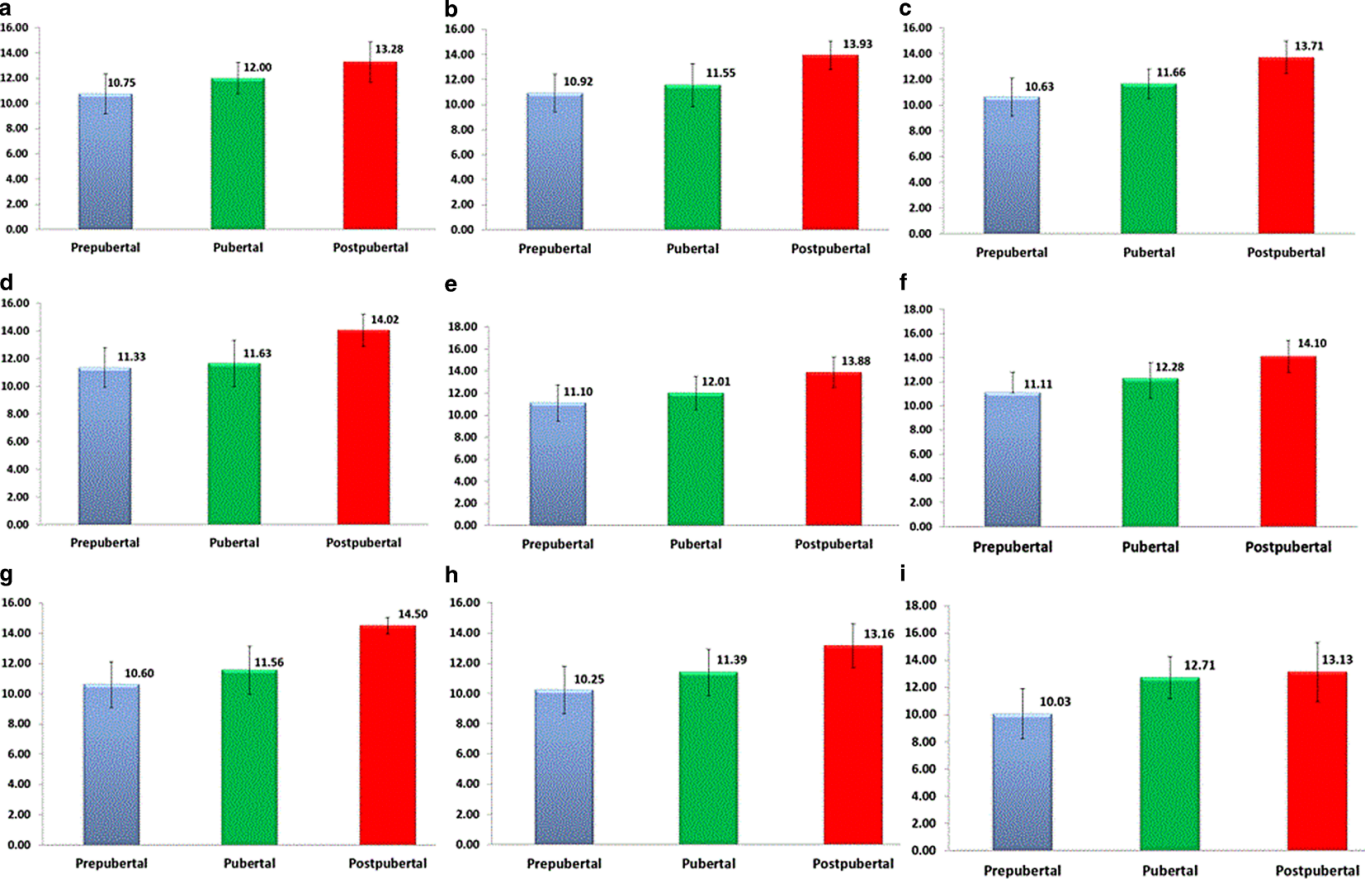
Bar charts showing the mean ages of the three growth stages in the nine skeletal growth patterns: **a** group 1 (CI, low), **b** group 2 (CI, normal), **c **group 3 (CI, high), **d **group 4 (CII, low), **e** group 5 (CII, normal), **f** group 6 (CII, high), **g **group 7 (CIII, low), **h **group 8 (CIII, normal), **i **group 9 (CIII, high) Balkendiagramme, die das mittlere Alter der 3 Wachstumsstadien in den 9 Skelettwachstumsmustern zeigen: **a** Gruppe 1 (CI, „low“), **b** Gruppe 2 (CI, normal), **c **Gruppe 3 (CI, „high“), **d **Gruppe 4 (CII, „low“), **e** Gruppe 5 (CII, normal), **f** Gruppe 6 (CII, „high“), **g **Gruppe 7 (CIII, „low“), **h **Gruppe 8 (CIII, normal), **i **Gruppe 9 (CIII, „high“)

Intragroup analysis (focused on testing the interval of time taken by each growth stage: prepubertal, pubertal and postpubertal). This was applied for all the 9 skeletal growth patterns (Tables 3 and 4; Fig. 3*). *It resulted in four groups (Table 5). The first group comprised skeletal patterns which demonstrated a statistically significant difference between the mean ages for the prepubertal and pubertal stages (i.e., had a delay in reaching their pubertal growth spurt): class II normal growers (*P* < 0.01), class II vertical growers (*P* < 0.001), class III normal growers (*P* < 0.01), and class III vertical growers (*P* < 0.001). The second group comprised the skeletal patterns not mentioned in the first group who reached their pubertal spurt earlier. The third group included skeletal patterns who demonstrated a statistically significant difference between the mean ages for the pubertal and postpubertal stages (i.e., had a prolonged pubertal stage): class I normal growers (*P* < 0.001), class I vertical growers (*P* < 0.01), class II horizontal growers (*P* < 0.01), class II normal growers (*P* < 0.001), class II vertical growers (*P* < 0.001), class III horizontal growers (*P* < 0.01), and class III normal growers (*P* < 0.01). The fourth group included the skeletal patterns not mentioned in the third group who had a short pubertal stage.

**Table 3:** Mean and standard deviation (SD) of the ages of the three growth stages (prepubertal, pubertal, postpubertal) within each skeletal growth pattern Mittelwert und Standardabweichung (SD) des Alters der 3 Wachstumsstadien (präpubertär, pubertär, postpubertär) innerhalb jedes skelettalen Wachstumsmusters

Skeletal growth pattern	Growth stage	Mean	Total Mean	SD	Total SD
*Class I low angle*	Prepubertal	10.75	12.2	1.58	1.84
	Pubertal	12		1.26	
	Postpubertal	13.28		1.6	
*Class I normal angle*	Prepubertal	10.92	11.72	1.48	1.87
	Pubertal	11.55		1.71	
	Postpubertal	13.93		1.12	
*Class I high angle*	Prepubertal	10.63	11.4	1.47	1.69
	Pubertal	11.66		1.16	
	Postpubertal	13.71		1.25	
*Class II low angle*	Prepubertal	11.33	12.58	1.41	1.86
	Pubertal	11.63		1.69	
	Postpubertal	14.02		1.16	
*Class II normal angle*	Prepubertal	11.1	11.85	1.62	1.83
	Pubertal	12.01		1.51	
	Postpubertal	13.88		1.37	
*Class II high angle*	Prepubertal	11.11	11.8	1.67	1.81
	Pubertal	12.28		1.3	
	Postpubertal	14.1		1.3	
*Class III low angle*	Prepubertal	10.6	12.2	1.52	2.04
	Pubertal	11.56		1.59	
	Postpubertal	14.5		0.55	
*Class III normal angle*	Prepubertal	10.25	10.94	1.56	1.82
	Pubertal	11.39		1.54	
	Postpubertal	13.16		1.45	
*Class III high angle*	Prepubertal	10.03	11.65	1.84	2.28
	Pubertal	12.71		1.55	
	Postpubertal	13.13		2.18	
*All groups*	Prepubertal	10.88	11.71	1.64	1.88
	Pubertal	11.97		1.49	
	Postpubertal	13.80		1.39

**Table 4:** Differences between the mean ages of the three growth stages within each skeletal growth pattern using ANOVA and Bonferonni methods for intragroup statistics Unterschiede zwischen den mittleren Alterswerten der 3 Wachstumsstadien innerhalb jedes skelettalen Wachstumsmusters unter Verwendung von ANOVA und Bonferonni-Methoden für Intragruppenstatistiken

Skeletal growth pattern	ANOVA	Bonferroni			
		Growth stage	Growth stage	Mean difference	P value
*Class I low angle (group 1)*	0.00534	Prepubertal	Pubertal	−1.25	0.43018
		Prepubertal	Postpubertal	−2.53	0.00430*
		Pubertal	Postpubertal	−1.28	0.32595
*Class I normal angle (group 2)*	0.00000	Prepubertal	Pubertal	−0.63	0.17119
		Prepubertal	Postpubertal	−3.01	0.00000*
		Pubertal	Postpubertal	−2.38	0.00000*
*Class I high angle (group 3)*	0.00001	Prepubertal	Pubertal	−1.04	0.05024
		Prepubertal	Postpubertal	−3.08	0.00001*
		Pubertal	Postpubertal	−2.05	0.00429*
*Class II low angle (group 4)*	0.00014	Prepubertal	Pubertal	−0.29	1.00000
		Prepubertal	Postpubertal	−2.69	0.00038*
		Pubertal	Postpubertal	−2.40	0.00199*
*Class II normal angle (group 5)*	0.00000	Prepubertal	Pubertal	−0.91	0.00267*
		Prepubertal	Postpubertal	−2.78	0.00000*
		Pubertal	Postpubertal	−1.87	0.00000*
*Class II high angle (group 6)*	0.00000	Prepubertal	Pubertal	−1.17	0.00000*
		Prepubertal	Postpubertal	−2.99	0.00000*
		Pubertal	Postpubertal	−1.82	0.00000*
*Class III low angle (group 7)*	0.00034	Prepubertal	Pubertal	−0.96	0.66333
		Prepubertal	Postpubertal	−3.90	0.00053*
		Pubertal	Postpubertal	−2.94	0.00204*
*Class III normal angle (group 8)*	0.00000	Prepubertal	Pubertal	−1.14	0.00502*
		Prepubertal	Postpubertal	−2.92	0.00000*
		Pubertal	Postpubertal	−1.78	0.00264*
*Class III high angle (group 9)*	0.00001	Prepubertal	Pubertal	−2.68	0.00010*
		Prepubertal	Postpubertal	−3.10	0.00017*
		Pubertal	Postpubertal	−0.42	1.00000

*Statistically significant

*ANOVA* analysis of variance

**Table 5:** Categorizing skeletal patterns according to the intragroup findings defining the cascade of their skeletal maturation Kategorisierung der skelettalen Muster nach den gruppeninternen Befunden, welche die Kaskade ihrer skelettalen Reifung definieren

Skeletal patterns with a delay reaching the pubertal growth spurt	Skeletal patterns who rapidly reached the pubertal growth spurt	Skeletal patterns having an extended pubertal growth period	Skeletal patterns having a short pubertal growth period
Class II N	Class I (H, N, V)	Class I (V, N)	Class I N
Class II V	Class II H	Class II (H, N, V)	Class III V
Class III N	Class III H	Class III (H, N)	
Class III V			

*N* normal, *V* vertical, *H* horizontal

**Fig. 4:**
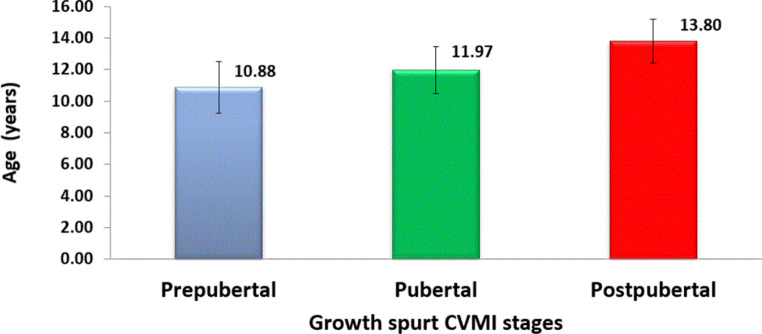
Bar chart showing the mean ages of the three growth stages in all studied subjects. *CVMI* cervical vertebrae maturation index Balkendiagramm mit dem mittleren Alter der 3 Wachstumsstadien bei allen untersuchten Probandinnen. *CVMI* „cervical vertebrae maturation index“

Numerical data defining the mean ages for the three growth stages in all the studied subjects is provided in Fig. 4.

## Discussion

The orthodontic literature embraces numerous attempts carried out to detect differences in skeletal maturation between different groups of studied subjects [12]. For instance, growing female subjects were reported to have an earlier pubertal growth spurt than growing males within the same age group. On the other hand, no difference in the pattern of skeletal maturation was found between subjects having tooth agenesis and unaffected controls [13].

Differences in skeletal maturation were detected between some skeletal configurations [7, 8]. Investigating longitudinal growth changes within a certain skeletal pattern has been done for some skeletal discrepancies such as for class II division 1 malocclusion [14]. Cross-sectional studies comparing the timing of growth within a certain skeletal discrepancy with that of a control class I group [7, 8] found that subjects with open and deep bites reached their pubertal growth at different time points [4].

The cervical vertebrae maturation index was used for assessing skeletal maturation, as it is an efficient tool and avoids the need for an extra radiation dose for a hand–wrist radiograph [15]. By using the 6 stages of the CVMI, it is possible to categorize the maturational data into three distinct growth stages.

It is noteworthy that previous reports focused only on the pubertal growth period [7, 8], where they included patients within stages 3 and 4 of the CVMI. The current research included in addition the prepubertal (CS1, CS2) postpubertal (CS5, CS6) stages. This aimed at defining a whole maturation map for all the possible skeletal anteroposterior and vertical combinations. Also, the present study included only female patients so as to exclude the gender difference confounding factor. Meanwhile, a parallel comparable study is being performed using male subjects.

The primary outcome data of the study aimed to analyze whether there was a difference between the onset of any of the three growth stages between the 9 skeletal growth patterns, while the secondary outcome data focused on determining the pattern of growth in each group by analyzing the time interval taken by each growth stage within each group. This intended to distinguish whether each growth stage lasted long or declined earlier within each group.

As for the primary outcome, no differences were found between the mean ages of the pubertal and postpubertal growth stages between the 9 skeletal patterns. For the prepubertal stage class III normal growers had an earlier onset reaching that stage when compared to that of class II high angle cases. This agrees with a recent systematic review that advocated early treatment for class III skeletal discrepancy [16].

**Fig. 5:**
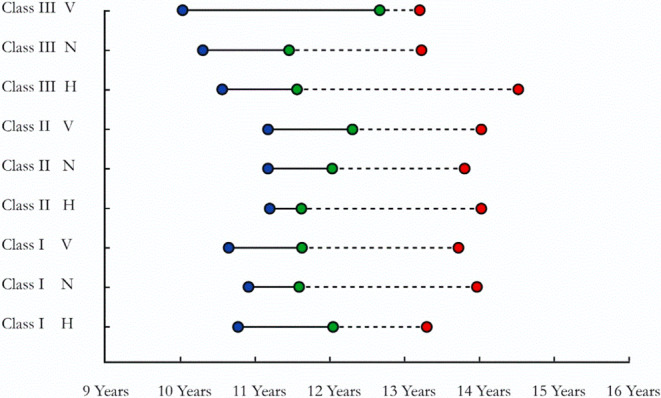
Chart showing the sequence of skeletal maturation in the 9 skeletal patterns. *Blue* prepubertal stage, *Green* pubertal stage, *Red* postpubertal stage Darstellung der Abfolge der Skelettreifung in den 9 skelettalen Mustern. *Blau* präpubertäres, *grün* pubertäres, *rot* postpubertäres Stadium

The secondary outcome focused on an intragroup analysis. The results revealed that the skeletal patterns could be classified into 4 categories (Table 5; Fig. 5). The first one comprised the skeletal patterns that demonstrated a delay in reaching the pubertal growth spurt. Class II normal and vertical growers and class III normal and vertical growers were included in this category. An interesting finding was that the vertical growth pattern of both the skeletal class II and class III subjects were included in this category. Subjects in this category would present a relatively long time interval staying in the prepubertal stage. Since maxillary growth modification was said to be better attained during the prepubertal stage [4], a considerable amount of time would be available for maxillary growth restriction or enhancement.

The second category comprised skeletal patterns that reached the pubertal growth spurt earlier denoting an early pubertal growth peak. In the subjects of this group, treatment protocols for early mandibular growth modification would be advisable [17].

The third group comprised skeletal patterns having a delayed transition from the pubertal period to the postpubertal growth stage denoting a prolonged pubertal growth period.

The fourth category included skeletal patterns having a rapid transition from the pubertal spurt interval to the postpubertal growth stage designating a short pubertal growth period. Class I low angle cases and class III high angle cases were present in this group. Accordingly, these patterns would offer a limited time for skeletal growth modification.

One of the attention-grabbing findings of this study was that the class III skeletal pattern as well as the horizontal and normal growing patterns demonstrated an extended pubertal growth period, while the vertical class III growers had a relatively short one. This confirms the necessity of offering a long retention period for class III adolescents having a horizontal growth pattern. Furthermore, skeletal class II cases with all vertical forms demonstrated an extended pubertal growth period, which would allow for a considerable time for mandibular growth modification.

According to the present results, it is evident that classifying growing patients based on their anteroposterior skeletal discrepancies solely is not sufficient, as within the same anteroposterior class, the vertical variants were found to follow variable growth sequences. Moreover, cases presenting with a skeletal class III exhibited an early pubertal growth spurt. This becomes clearly obvious when looking at the chart shown in Fig. 5, which confirms the long-established recommendation to start treating class III skeletal discrepancy cases early in the prepubertal stage.

In summary, each skeletal pattern should receive growth modification treatment at different time intervals with inequivalent starting and ending time points (Fig. 5). Information regarding the age at which each group reaches the prepubertal and pubertal growth stages could help the clinician to be more competent in customizing an individualized growth modification protocol for each skeletal growth pattern. Moreover, the study of the skeletal maturation elucidated an average 3‑year growth period for all the studied skeletal groups. This gives an indication of the limited time period within which growth modification could be achieved.

## Conclusions

- Class III normal growers demonstrated an earlier onset of prepubertal growth stage when compared to class II vertical growers.
- No statistically significant differences were detected regarding the onset of both the pubertal and postpubertal stages of the 9 skeletal patterns.
- The vertical growing patterns of class II and class III patients demonstrated a delay in reaching the pubertal growth spurt, while the horizontal growers of the same classes had an early pubertal spurt onset.
- Class III horizontal and normal growers demonstrated an extended pubertal growth period, while the vertical growers had an earlier pubertal growth spurt.
